# Enhancing Selectivity with Molecularly Imprinted Polymers via Non-Thermal Dielectric Barrier Discharge Plasma

**DOI:** 10.3390/polym16162380

**Published:** 2024-08-22

**Authors:** Samira Amiri Khoshkar Vandani, Qianwei Liu, Yuki Lam, Hai-Feng Ji

**Affiliations:** Department of Chemistry, Drexel University, Philadelphia, PA 19104, USA; sa3948@drexel.edu (S.A.K.V.); ql88@drexel.edu (Q.L.); ml3593@drexel.edu (Y.L.)

**Keywords:** molecularly imprinted polymer, MIP, non-thermal dielectric barrier discharge plasma, DBD polymer, polymerization, protein identification, man-made antibody

## Abstract

Molecularly imprinted polymers (MIPs) are synthetic polymers that mimic the functions of antibodies. Though MIPs are promising tools in various areas, achieving high selectivity in MIPs can be difficult. To improve selectivity, various approaches have been implemented; however, the role of polymerization methods or synthetic techniques in enhancing the selectivity of MIPs has not been studied and remains a crucial area for further research. MIPs are typically prepared from free radical reactions. Recently, we found that Dielectric Barrier Discharge (DBD) plasma can be used to initiate the polymerization of vinyl monomers. The DBD plasma method allows the monomers to associate with the template molecules and initiate polymerization with minimal disruption to the positioning of the monomers. We hypothesize that this could be a preferred method to prepare MIPs over the traditional radical reaction that may cause a disturbance of the pre-associated monomers on the templates for the polymerization. Chicken egg white serum albumin (CESA) was used as the template protein for the MIPs. Our results show that in all test conditions, approximately twofold improvement in selectivity was achieved, which is the primary performance metric for MIPs. This enhancement was evident across all categories, including MIPs prepared from various monomer combinations.

## 1. Introduction

Molecularly imprinted polymers (MIPs) are synthetic polymers designed to have specific recognition sites for target molecules, enabling them to bind selectively to specific substrates, similar to natural receptors or antibodies. They are typically used for the detection, isolation, and separation of the target molecules. The preparation of MIPs involves three main steps: assembling target molecules as template with functional monomers, polymerizing the monomers and crosslinkers around the templates to form a polymer matrix, and removing the template molecules to leave behind cavities that match the shape, size, and functional groups of the template molecules [1]. Monomers play an essential role in the production of MIPs, as their selection determines the ability to organize functional groups during assembly [2]. During polymerization, the monomers and crosslinkers form a polymer matrix around the template molecules. After the polymer matrix forms, the template molecules are removed, leaving behind cavities that match the shape, size, and functional groups of the template [3]. These fine-tuned cavities allow MIPs to rebind template molecules with high selectivity through specific interactions such as hydrogen bonds, van der Waals forces, and ionic bonds. MIPs offer several advantages over natural receptors and antibodies: they are stable, reusable, affordable, and, most importantly, highly selective for template molecules [4]. Unlike natural receptors and antibodies, which can be easily degraded by enzymatic activities or environmental factors, MIPs are thermally and chemically robust [5]. Additionally, they can be stored for long periods without losing their binding capability, unlike natural antibodies [6]. The synthesis of MIPs is more cost-effective than that of natural antibodies since it does not involve complex biological processes [7]. Furthermore, MIPs’ robust structure and environmental stability allow them to be reused without significant loss of binding efficiency and selectivity [8]. Due to these properties, MIPs are promising for a wide range of applications, including separation and purification, sensors and biosensors, drug delivery, and catalysis.

Though MIPs are promising tools in various areas, offering customizable selectivity and reusability, they face challenges such as template retention, template leakage, and the rupture of the polymer matrix [9]. Most importantly, achieving high selectivity in MIPs can be difficult despite the imprinting process introducing binding-specific cavities [10]. To improve selectivity, various approaches have been implemented, including the selection of monomers, effective template removal, and the protection of cavities from collapsing or distortion [11]. However, the role of polymerization methods or synthetic techniques in enhancing the selectivity of MIPs and the efficiency of their synthesis has not been studied and remains a crucial area for further research.

**Figure 1:**
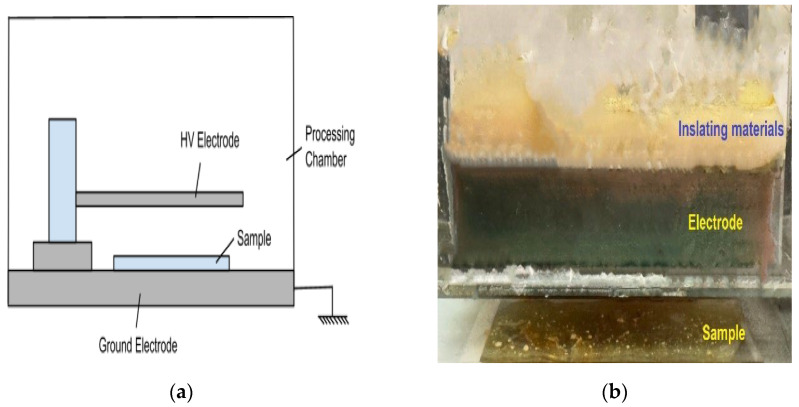
(**a**) Scheme of a DBD plasma setup. (**b**) The DBD electrode was made of a 38 mm × 64 mm copper plate covered with a 1 mm-thick glass strip. The discharge gap for plasma was 5 mm. Plasma was observed on a glass slide between the two insulated electrodes.

MIP are typically prepared from free radical chain-growth reaction [12]. The radical chain-growth polymerizations are initiated by free radicals that are generated from the thermal, photo, or redox decomposition of initiator species. Recently, we found that Dielectric Barrier Discharge (DBD) plasma can be used to initiate the polymerization of both vinyl and heterocyclic monomers [13]. DBD plasma is a non-thermal plasma generated under ambient conditions (i.e., room temperature and atmospheric pressure). It is produced by applying a high voltage between the electrodes [14] and is therefore sometimes called ‘cold plasma’ to highlight this distinction from hot plasma [15,16]. Either one of the two electrodes or both are covered with an insulating material to attenuate electron flow between the two electrodes (Figure 1a). The dielectric insulator prevents a build-up of high currents between the electrodes, creating an electrically safe plasma without significant gas heating. This makes DBD energy-efficient and non-thermal. (Figure 1b). When generated in air, plasma contains reactive oxygen and nitrogen species (ROS and RNS, respectively) including ozone (O_3_), superoxide anion (O_2_^−^), nitric oxide (•NO), hydroxyl radical (•OH), and electrons [13,17,18,19,20,21,22,23,24] which can initiate the polymerization reaction. Thus, plasma can be applied on samples in situ without much disturbance of the samples.

This method to prepare polymerization products is different from the traditional gaseous plasma polymerization method [25]. We would term this method “DBD Non-Gaseous Polymerization (DBD NGP)”, but will simply describe the method as ‘DBD plasma polymerization’ in this work. It is complementary to the former method since it includes monomers in both the solid and liquid states. The DBD plasma approach has the advantage of being more energy-efficient than the hot gaseous plasma method because a larger portion of energy in DBD is converted into the production of various oxidative species instead of heating the entire gas stream. More importantly, the DBD NGP method allows the monomers to associate with the template molecules and initiate polymerization with minimal disruption to the positioning of the monomers. This may result in the formation of highly efficient and selective binding cavities in the MIPs. We hypothesize that this could be a preferred method to prepare MIPs over the traditional radical reaction that may cause a disturbance of the pre-associated monomers on the targets for the polymerization of monomers in MIP production. 

The main goal of this work is to validate the DBD plasma methodology for the synthesis and performance study of MIP materials. However, the elimination of chicken egg serum albumin (CESA) is also important. In the food industry, CESA is a significant allergen, necessitating detection and removal procedures to prevent allergic reactions in sensitive individuals. This approach avoids cross-contamination and ensures consumer safety in food production. In biotechnology and pharmaceuticals, it is crucial to correctly eliminate CESA from biologics, such as vaccines, to prevent potential reactions in patients, especially those with egg allergies [26]. The accurate detection of CESA is also essential for identifying potential contamination and evaluating allergenic risks, making it crucial for diagnostic assays [27,28].

## 2. Materials and Methods

### 2.1. Materials

Chicken egg white serum albumin (CESA), bovine serum albumin (BSA), 2-methacryloyloxyethyl phosphoryl choline (MPC), and methacrylic acid were purchased from Sigma (St. Louis, MO, USA); Ammonium persulfate (APS) was obtained from Fisher (Ottawa, ON, USA); N,N,N′,N′-tetramethylethylenediamine (TEMED), N,N′-methylenebisacrylamide (BIS), 4-methyl-5-vinylthiazole, 3-ethyl-1-vinyl-1H-imidazol-3-ium bromide, arylamide, dimethyl aminoethyl methacrylate (DMAEMA), 1-(3-Sulfopropyl)-2-vinylpyridinium hydroxide, 4-vinylbenzoic acid, N-vinylformamide, 1-vinylimidazole, and 4-vinylphenyl acetate, 4-vinylphenylboronic acid, and 2-Methacryloyloxyethyl phos-phorylcholine (MPC) were purchased from TCI America (Portland, OR, USA). The monomers used in this work are shown in Table 1.

### 2.2. MIP Synthesis Using a Traditional Radical Reaction Process

In total, 0.2 g of CESA was dissolved in 5 mL of phosphate buffer (20 mM, pH 5.5). Then, 0.06 g of monomers and 0.018 g of crosslinker BIS were added to the solution. The mixture was purged with nitrogen for 15 min. After purging, 75 µL of APS (10% *v*/*v*) and 25 µL of TEMED were immediately added to the mixture. The solution was incubated for 8 h. The resulting material was ground with a mortar and pestle until it became a fine powder, preparing it for the template removal process. The mass of monomers that were used for each group is listed in Table 2.

### 2.3. DBD Plasma Generation

DBD air plasma was generated using a microsecond-pulsed power supply (FID Technology, Burbach, Germany) and an electrode dielectric-barrier discharge setup, as shown in Figure 1. The DBD electrode creates a plasma stream between a high-voltage 25 mm thick copper plate and the ground. A 1 mm thick quartz dielectric plate serves as an insulating barrier, covering the copper plate. The plasma discharge gap between the bottom of the quartz plate and the surface of the samples was maintained at 5 mm. Plasma was generated using a variable voltage and frequency power supply, applying a pulsed alternating polarity voltage of 20 kV (peak-to-peak) with a 10 ns pulse width and a rise time of 5 V/ns. For all experiments, a peak voltage of 11.2 kV and a repetition frequency of 690 Hz were used. The input energy was calculated to be approximately 10 mJ per pulse. The working area for plasma treatment corresponded to the copper plate dimensions of 38 mm × 64 mm.

### 2.4. MIP Synthesis Using the DBD Plasma Method

In total, 0.2 g of CESA was dissolved in 1 mL of phosphate buffer (pH 5.5). Next, 0.06 g of monomers and 0.018 g of BIS were added to the solution. The mixture was drop-casted onto a glass slide and exposed to DBD plasma for 15 min. The solid materials after the plasma treatment were insoluble in any solvents, so they were scraped from the glass plate and ground into a fine powder. The mass of monomers that were used for each group is listed in Table 2.

### 2.5. Removal of the CESA Template from the MIPs

The MIPs were washed with phosphate buffer (pH 5.5) for 12 h to remove all the templates. Following this, the MIPs were rinsed and equilibrated with deionized water, and then dried at room temperature for 24 h.

### 2.6. Rebinding of CESA Using the MIPs

A 1 mg/mL solution of CESA was prepared in phosphate buffer (pH 5.5). To this solution, 1 mg of MIPs was added. The mixture was vortexed for various durations, followed by centrifugation for 5 min to separate the MIPs. The UV-Vis spectra of the solutions were recorded to determine the binding efficiency of CESA.

The CESA binding efficiency of the prepared MIPs after 6 h was calculated using Equation (1):

Efficiency = (C_i_ − C_f_/C_i_) × 100 (1)

where C_i_ and C_f_ are the initial and final concentration of template CESA protein in buffer solution, measured at 278 nm, respectively.

### 2.7. Rebinding of BSA and Selectivity towards CESA Using the MIPs

A 1 mg/mL solution of BSA was prepared in phosphate buffer (pH 5.5). To this solution, 1 mg of MIPs was added. The mixture was vortexed for 6 h, followed by centrifugation for 5 min to separate the MIPs. The UV-Vis spectra of the solutions were recorded to determine the binding efficiency of BSA and the selectivity of the MIPs towards CESA compared to BSA.

The selectivity towards CESA binding over BSA was calculated using Equation (2):

Selectivity = Binding efficiency of template molecules (CESA)/nonspecific binding efficiency of BSA(2)

### 2.8. Characterization

Since the MIPs are insoluble network polymers, FTIR (Fourier-Transform Infrared) was used to characterize MIPs prepared using both traditional and plasma polymerization methods, both before and after the removal of the template protein. FTIR spectroscopy is a reliable and cost-effective analytical tool for determining the functional groups of polymers. 

## 3. Results and Discussion

CESA was used as the template for the MIPs, while BSA was utilized as a control for nonspecific binding to calculate selectivity. The efficiency and selectivity of MIPs prepared using traditional radical reactions were measured and set as the baseline for comparison. 

**Figure 2:**
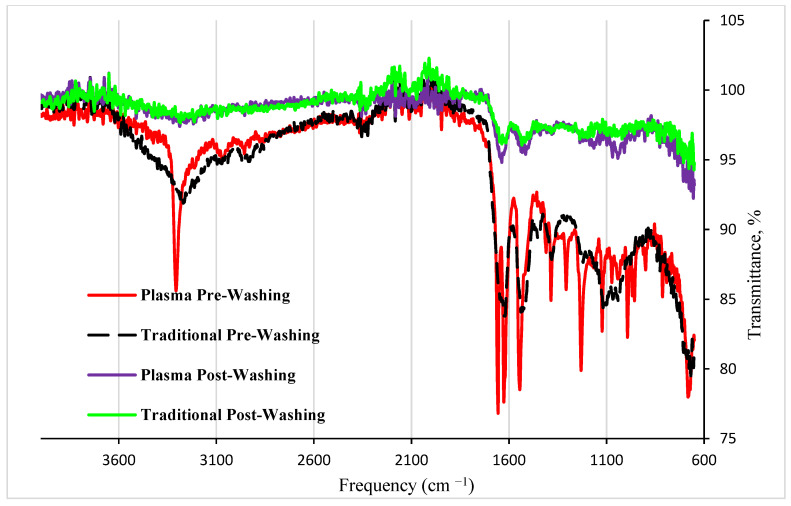
FTIR spectra of MIPs synthesized from the traditional radical reactions and the DBD plasma using the combination of six monomers before and after washing to remove CESA.

To confirm the synthesis of polymers using the DBD plasma method, FTIR spectra of MIPs synthesized with six different monomers under DBD plasma were collected and compared with those produced using the traditional radical method, as shown in Figure 2. The results in Figure 2 indicate that the polymers prepared using the DBD plasma method are intrinsically the same as those synthesized via radical reactions, both before and after washing to remove CESA. The common peaks and their corresponding contributions are listed and discussed below:

1. The most characteristic peaks were observed at 1657 cm^−1^, corresponding to the amide I band (–C=O stretching), and at 1533 cm^−1^, corresponding to the amide II band (C-N stretch with N-H bending). These peaks significantly decreased after washing, indicating that most of these peaks are from the CESA protein, so the peaks diminished when CESA is removed from the MIP cavities. Additionally, the appearance of sharp peaks at 3300 cm^−1^ from the MIP prepared from DBD plasma, which was not observed from that prepared from traditional radical reactions, indicates more OH or NH groups in this MIP, which is the major difference between the two polymers. It is known that these groups can be generated in DBD plasma. The peak at around 890 cm^−1^ can be associated with the C-C stretching vibration in the polymer backbone.
2. The peaks around 1040–1100 cm^−1^ indicate the presence of S=O stretching from sulfonate groups in the 1-(3-sulfopropyl)-2-vinylpyridinium monomer. The peaks around 1700–1725 cm^−1^ are attributed to the carboxylic acid in 4-vinylbenzoic acid. The peaks around 1735–1750 cm^−1^ are attributed to the ester C=O in 4-vinylphenyl acetate. The peaks around 1500–1600 cm^−1^ are due to the benzene rings in several monomers. The peaks at 3589–3602 cm^−1^ correspond to the O-H or N-H stretching vibrations. These peaks indicate that all six monomers have been involved in the polymerization.

**Figure 3:**
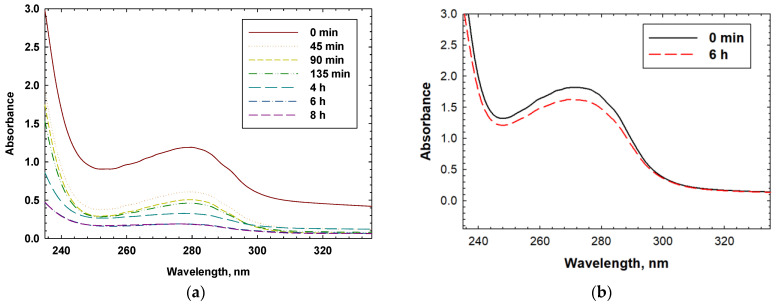

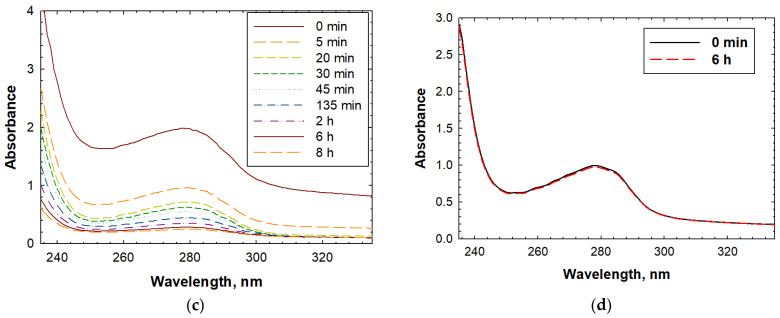
(**a**) UV-Vis spectrum of CESA upon binding with an MIP synthesized using the combination of six monomers and radical polymerization methodology. (**b**) UV-Vis spectrum of BSA upon binding with an MIP synthesized using the combination of six monomers in Table 3 and radical polymerization methodology. (**c**) UV-Vis spectrum of CESA upon binding with an MIP synthesized using the combination of six monomers and plasma polymerization methodology. (**d**) UV-Vis spectrum of BSA upon binding with an MIP synthesized using the combination of six monomers in Table 3 and plasma polymerization methodology.

Figure 3 present typical UV spectra of MIPs binding with CESA and the interaction of MIPs with BSA for nonspecific binding studies. All MIPs showed a preference for binding CESA over BSA. The binding efficiencies for CESA and BSA, as well as the calculated selectivity, are summarized in Table 3.

According to MIP theory, a single monomer is insufficient for preparing an efficient MIP [29]. A combination of two or more complementary monomers is needed, such as those with positive and negative charges, to interact with various sites of a protein before polymerization, creating a synergistic effect.

Table 3 shows that two similar monomers do not yield efficient MIPs. For example, using two acidic monomers, such as 4-vinylbenzoic acid and 4-vinylphenylboronic acid, resulted in low efficiency (68%) and relatively poor selectivity (3.4). Similarly, two neutral species, such as N-vinylformamide and 4-vinylphenyl acetate, also exhibited low efficiency (67%) and poor selectivity (3.5). The combination of one negatively charged and one positively charged species, such as 4-vinylbenzoic acid and 3-ethyl-1-vinyl-H-imidazol-3-ium bromide, showed an improvement in efficiency and selectivity (74% and 4.6, respectively). Further improvement was observed with the combination of three species with varying properties, such as N-vinylformamide, 4-vinylbenzoic acid, and 3-ethyl-1-vinyl-H-imidazol-3-ium bromide, achieving 71% efficiency and 6.5 selectivity. More significant improvements (85% efficiency and 12.1 selectivity) were observed when six monomers with diverse properties were used: 1-(3-sulfopropyl)-2-vinylpyridinium, 4-vinylbenzoic acid, N-vinylformamide, 1-vinylimidazole, 4-vinylphenyl acetate, and 4-vinylphenylboronic acid. These results are consistent with publications showing that multi-monomer MIPs create a synergistic effect, enhancing both efficiency and selectivity [30]. The synergistic effect significantly contributes to higher selectivity by favoring the formation of multiple interactions—such as hydrogen bonds, electrostatic attraction, polar attraction, and van der Waals interactions—between the functional monomers and the template protein during the preparation of MIPs. This increased diversity of functional sites enhances the interaction between the template protein and the MIPs, thereby improving the efficiency of the imprinting process.

**Figure 4:**
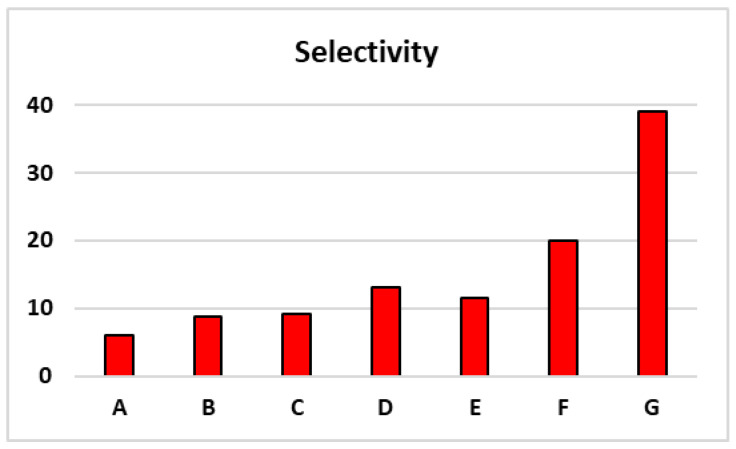
Comparison of selectivity among A to G monomer combinations listed in Table 3.

The above results set the baseline for comparison with MIPs made using the plasma method. Table 3 also compares the efficiency and selectivity of MIP polymers prepared using the traditional radical method versus the DBD plasma method. While the efficiency showed minimal change, the nonspecific binding to BSA significantly decreased across all categories, leading to a substantial improvement in selectivity—the primary performance metric for MIPs prepared using the DBD plasma method, which improved by approximately more than twofold. This enhancement was evident across all categories, including MIPs prepared from two acidic monomers like 4-vinylbenzoic acid and 4-vinylphenylboronic acid, which exhibited a selectivity of 6% compared to 3.4% for MIPs from radical reactions. For MIPs prepared from six monomers with diverse properties, namely, 1-(3-sulfopropyl)-2-vinylpyridinium, 4-vinylbenzoic acid, N-vinylformamide, 1-vinylimidazole, 4-vinylphenyl acetate, and 4-vinylphenylboronic acid, the selectivity was 20%, a near two-fold higher than the 12.1% observed in MIPs from radical reactions. The addition of 2-Methacryloyloxyethyl phos-phorylcholine (MPC) in the above six monomers resulted in a 39% selectivity, significantly higher than the 11.9% observed in MIPs from radical reactions. The role of MPC to significantly enhance the selectivity is not fully understood yet, but the result demonstrated that the major impact on the substantial improvement in selectivity is through the lowering of the nonspecific binding to BSA (2%) as comparison to that from the traditional radical polymerization (7%). Figure 4 presents a selectivity comparison of various monomer combinations for a straight demonstration. 

## 4. Conclusions

To improve selectivity of molecularly imprinted polymers (MIPs), we synthesized MIPs using a non-thermal, dielectric barrier discharge (DBD) plasma method for the target protein chicken egg white serum albumin (CESA) and compared the selectivity of the MIPs with those prepared using the traditional radical method. MIPs made of several combinations of monomers with diverse properties were studied. The result showed a two-to-three-fold improvement in selectivity, the primary performance metric for MIPs, across all monomer combinations and test conditions when using the DBD plasma method compared to the MIPs from the traditional radical reactions. The higher selectivity may be due to the minimal disturbance of the pre-associated monomers on the targets for the polymerization of monomers in MIP production under DBD plasma, while the traditional radical reaction causes more disturbance of the pre-associated monomers on the targets because of heat.

This work takes advantage of the recent development of several new kinds of plasma devices generating DBD plasma which have extended plasma’s potential applications [31,32,33]. The DBD plasma polymerization technique has several advantages for radical polymerization in industrial settings [34,35]. It is lower in cost [36], less polluting to the environment due to the absence of radical initiators, and requires short treatment times [37]. More importantly, the method showed distinct advantages of DBD plasma and was the preferred method over the traditional radical methods in MIP formation conditions, showing a better selectivity as demonstrated in our experiments. 

Since this is the first ever and initial report on MIP preparation using the DBD plasma method, the utilization of DBD plasma for polymerization to create MIPs remains insufficiently explored, constraining the method’s applications. For proof of concept of the DBD plasma method, we selected monomers with various functional groups, such as COOH, benzene rings, ammonium, amide, and anions, to diversify the functional groups. However, the combination of functional monomers must be carefully selected and extensively tested in the future to develop MIPs with superior characteristics. Future studies will focus on optimizing the synergistic effect of the MIP combination. Ongoing research in our laboratory aims to address this gap by investigating various aspects such as monomer selection, reaction duration, conditions, and thorough polymer characterization. These efforts seek to establish optimal conditions and provide guidance on employing plasma-based methods for MIP preparation, thereby assessing its ultimate potential promise.

## Figures and Tables

**Table 1:** Names, structures, and properties of monomers.

Name	Structure	Properties
1-(3-Sulfopropyl)-2-vinylpyridinium Hydroxide Inner Salt	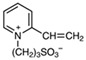	Has both negative and positive components
4-Vinylbenzoic Acid	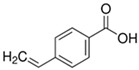	Contains a COOH for hydrogen bonding
4-Vinylphenylboronic Acid	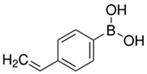	Contains a COOH for hydrogel bonding
3-Ethyl-1-vinyl-H-imidazol-3-ium bromide	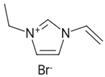	Has a positive charge
4-Methyl-5-vinylthiazole	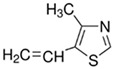	Has a positive charge
1-Vinylimidazole	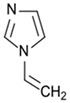	Positive in pH 5.5 buffer solution
N-Vinylformamide	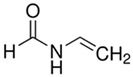	Electrically neutral
4-Vinylphenyl Acetate	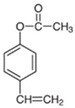	Electrically neutral
2-Methacryloyloxyethyl phosphorylcholine	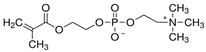	Contains both positive and negative charges

**Table 2:** The mass of monomers used for each type of MIPs.

Monomer Names	Mass of Monomers Used (g)
4-Vinylbenzoic Acid	0.03
4-Vinylphenylboronic Acid	0.03
N-Vinylformamide	0.03
4-Vinylphenyl Acetate	0.03
4-Vinylbenzoic Acid	0.03
3-Ethyl-1-vinyl-H-imidazol-3-ium bromide	0.03
N-Vinylformamide	0.03
1-(3-Sulfopropyl)-2-vinylpyridinium	0.03
N-Vinylformamide	0.03
4-Vinylbenzoic Acid	0.03
N-Vinylformamide	0.015
4-Vinylphenylboronic Acid	0.015
3-Ethyl-1-vinyl-H-imidazol-3-ium Bromide	0.015
4-Methyl-5-vinylthiazole	0.015
N-Vinylformamide	0.02
4-Vinylbenzoic Acid	0.02
3-Ethyl-1-vinyl-H-imidazol-3-ium bromide	0.02
4-Vinylbenzoic Acid	0.02
4-Methyl-5-vinylthiazole	0.02
1-Vinylimidazole	0.02
1-(3-Sulfopropyl)-2-vinylpyridinium	0.01
4-Vinylbenzoic Acid	0.01
N-Vinylformamide	0.01
1-Vinylimidazole	0.01
4-Vinylphenyl Acetate	0.01
4-Vinylphenylboronic Acid	0.01
1-(3-Sulfopropyl)-2-vinylpyridinium	0.01
4-Vinylbenzoic Acid	0.01
N-Vinylformamide	0.01
1-Vinylimidazole	0.01
4-Vinylphenyl Acetate	0.01
4-Vinylphenylboronic Acid	0.01
2-Methacryloyloxyethyl phosphorylcholine	0.01

**Table 3:** The efficiency and selectivity of MIPs prepared using the traditional and DBD plasma polymerization methods.

	Traditional Radical Polymerization			DBD Plasma Polymerization		
Monomers	Efficiency	Nonspecific Binding	Selectivity	Efficiency	Nonspecific Binding	Selectivity
4-Vinylbenzoic Acid 4-Vinylphenylboronic Acid (A)	68%	20%	3.4	72%	12%	6
					
					
N-Vinylformamide 1-(3-Sulfopropyl)-2-vinylpyridinium (B)	79%	17%	4.6	80%	9%	8.8
					
N-Vinylformamide 4-Vinylphenylboronic Acid 3-Ethyl-1-vinyl-H-imidazol-3-ium Bromide 4-Methyl-5-vinylthiazole (C)	62%	12%	5.1	65%	7%	9.2
					
N-Vinylformamide 4-Vinylbenzoic Acid 3-Ethyl-1-vinyl-H-imidazol-3-ium Bromide (D)	71%	11%	6.5	78%	6%	13
					
4-Vinylbenzoic Acid 4-Methyl-5-vinylthiazole 1-Vinylimidazole (E)	63%	11%	5.72	81%	7%	11.6
					
1-(3-sulfopropyl)-2-vinylpyridinium 4-vinylbenzoic acid N-vinylformamide 1-vinylimidazole 4-vinylphenyl acetate 4-vinylphenylboronic acid (F)	82%	7%	12.1	88%	4%	20
					
2-Methacryloyloxyethyl phosphorylcholine 1-(3-sulfopropyl)-2-vinylpyridinium 4-vinylbenzoic acid N-vinylformamide 1-vinylimidazole 4-vinylphenyl acetate 4-vinylphenylboronic acid (G)	83%	7%	11.9	78%	2%	39
					

## Data Availability

Data are contained within the article.
